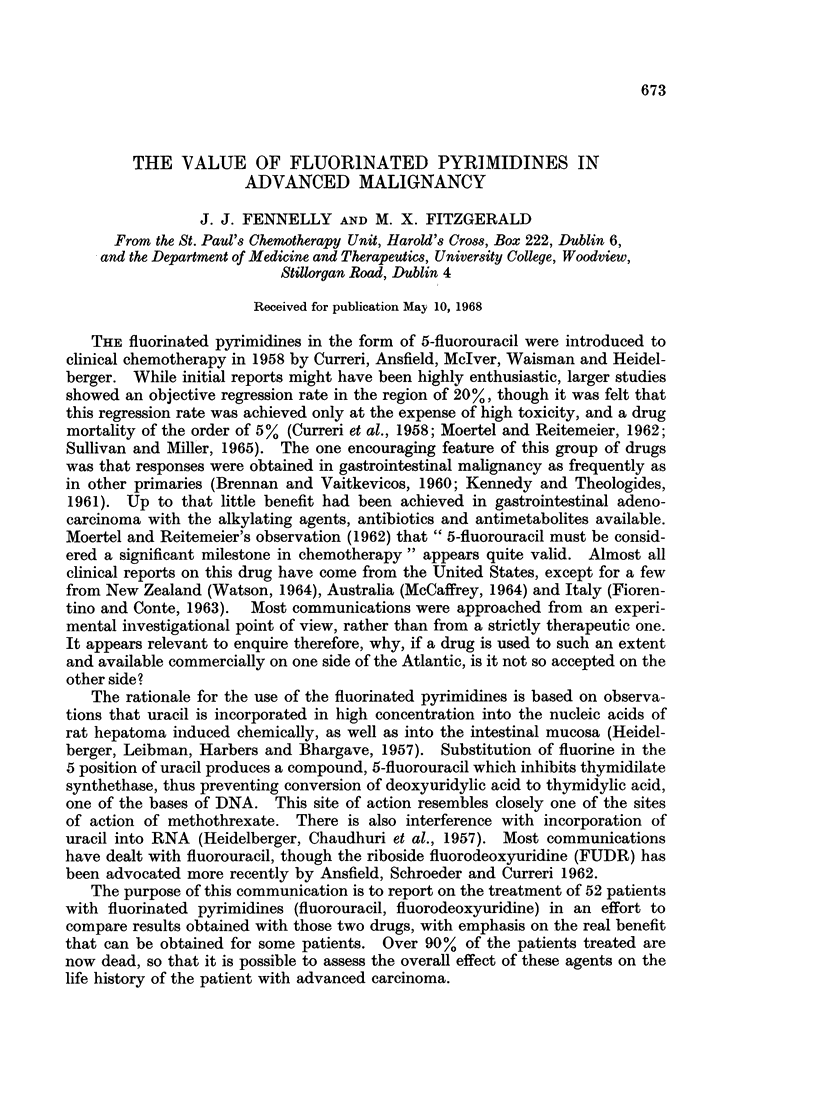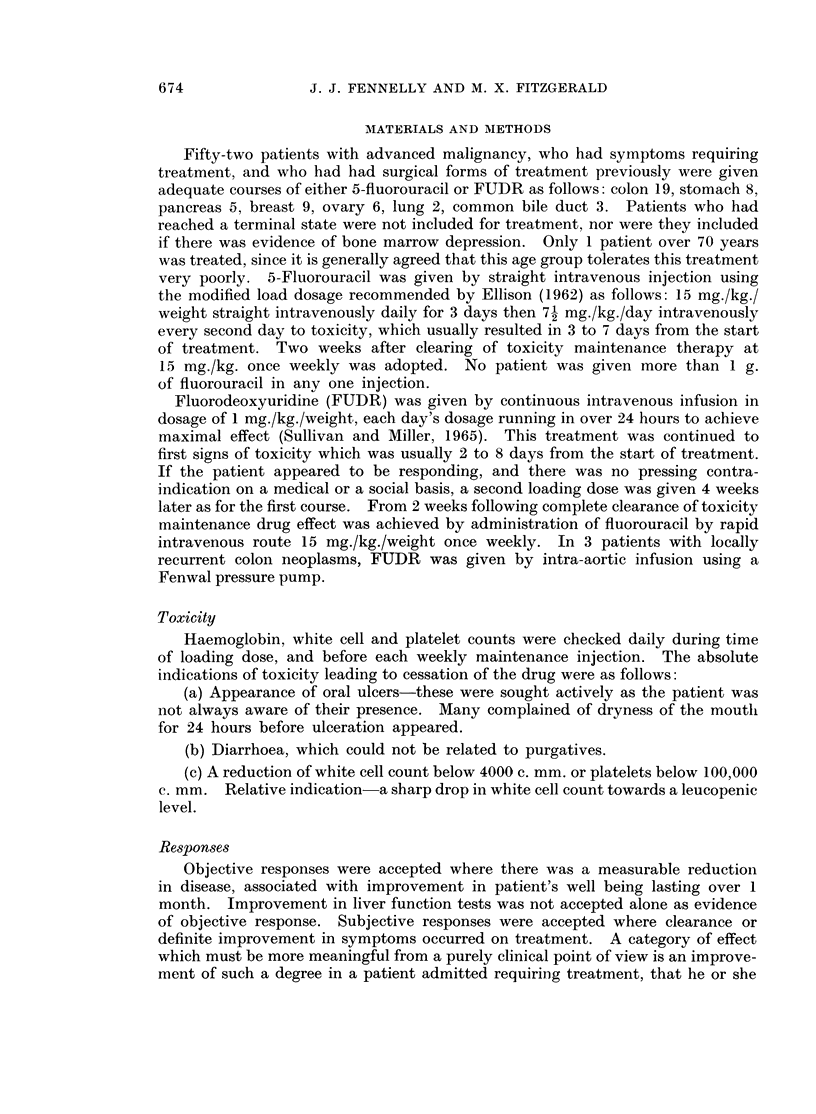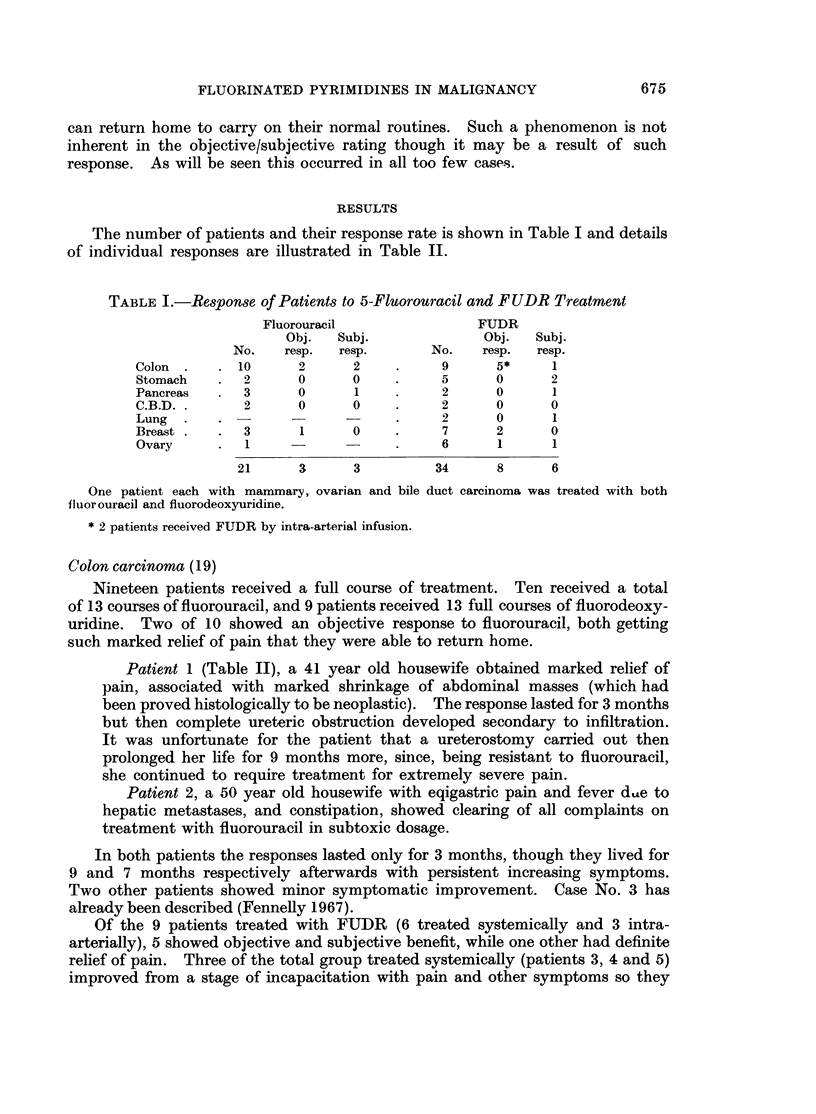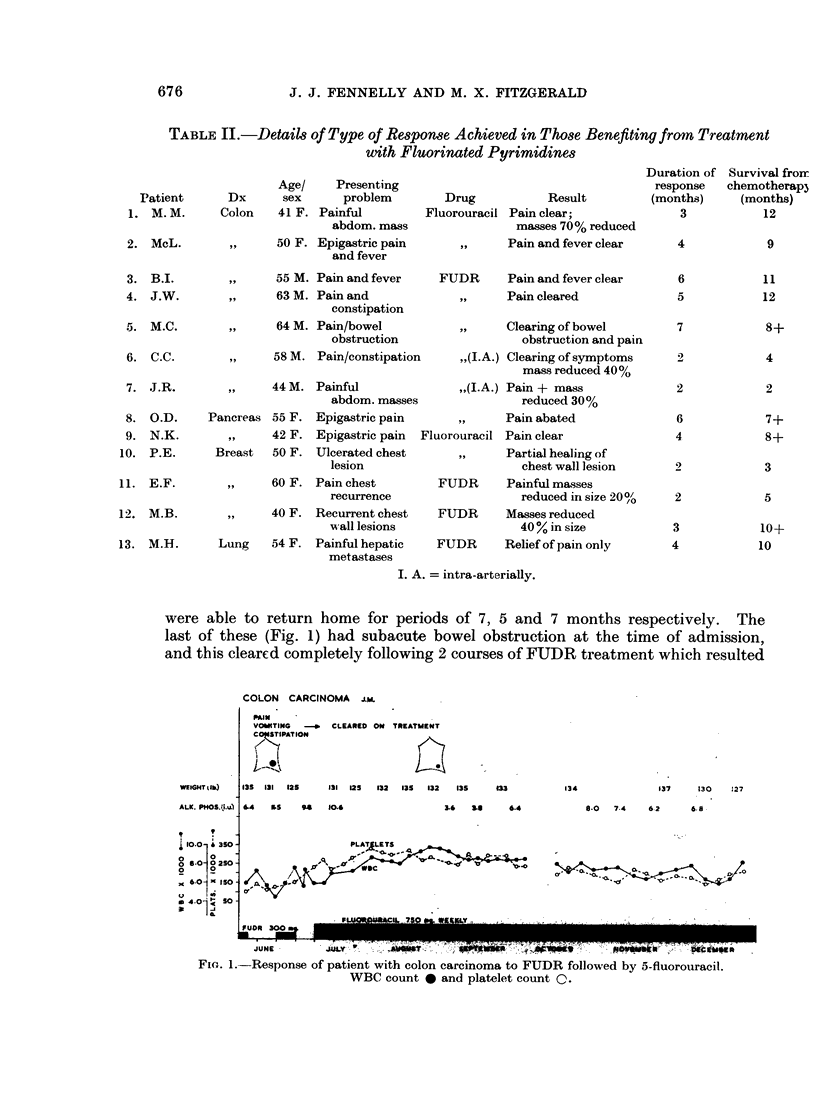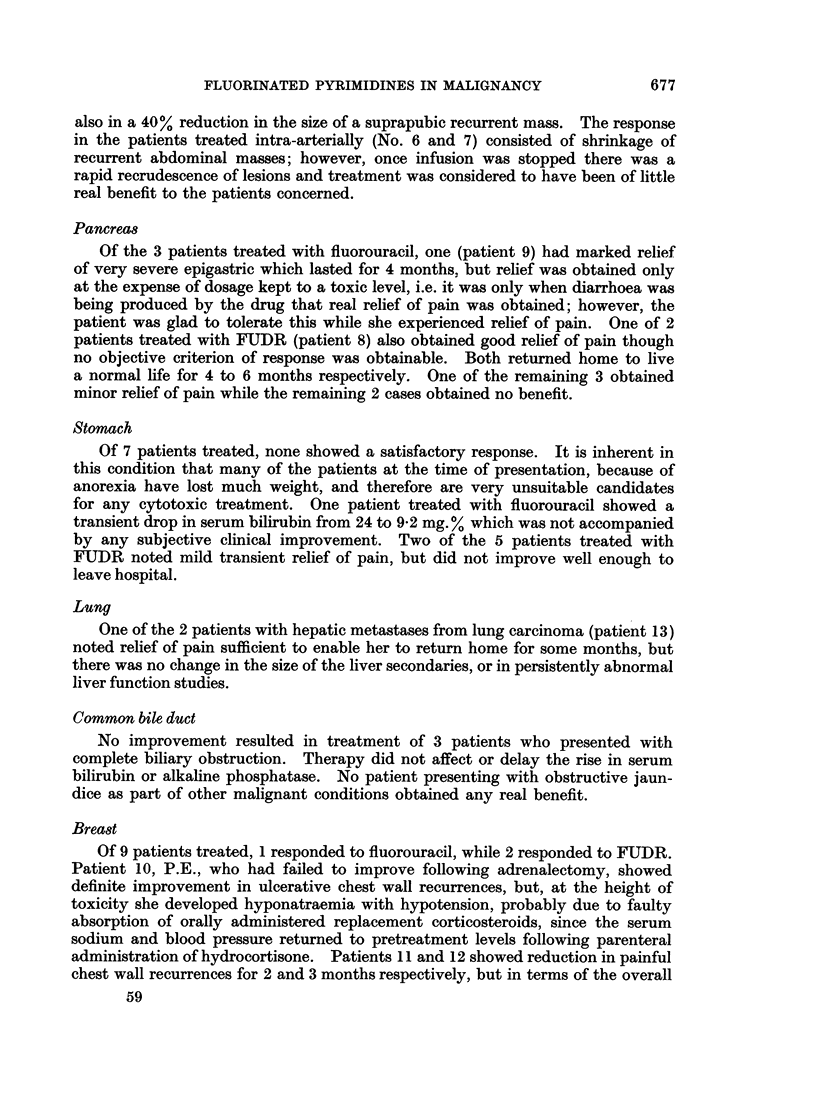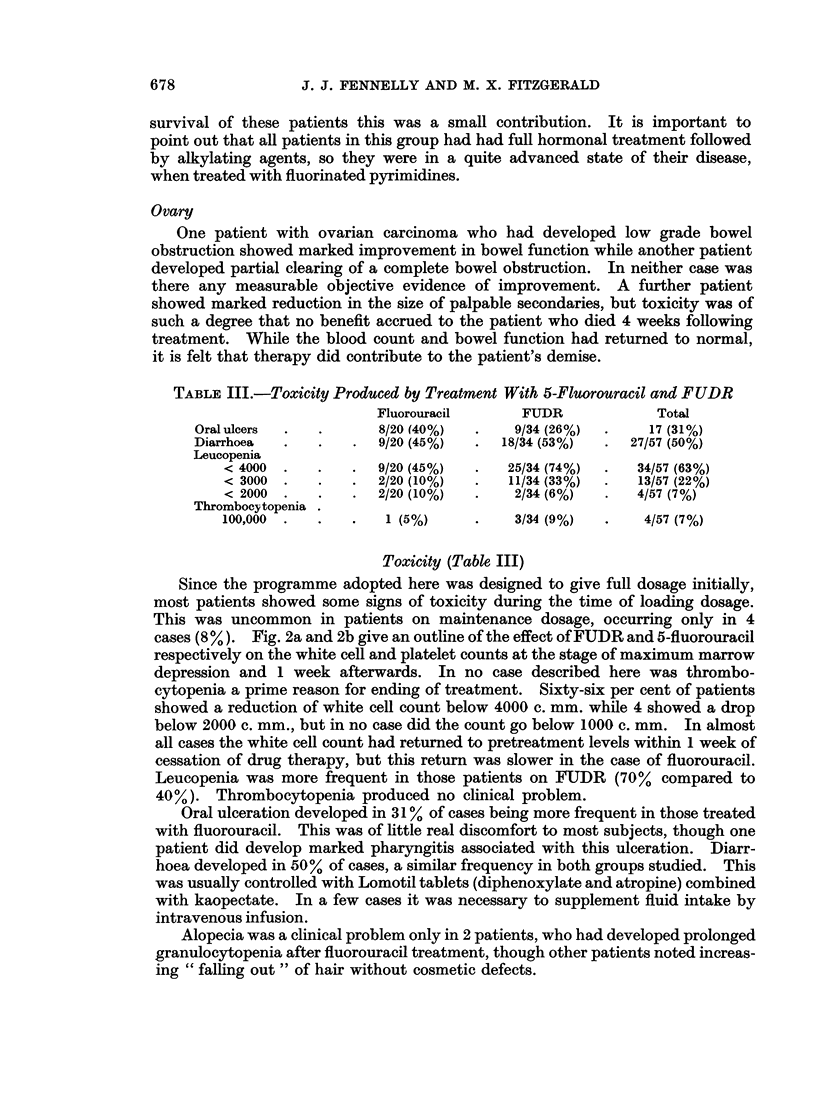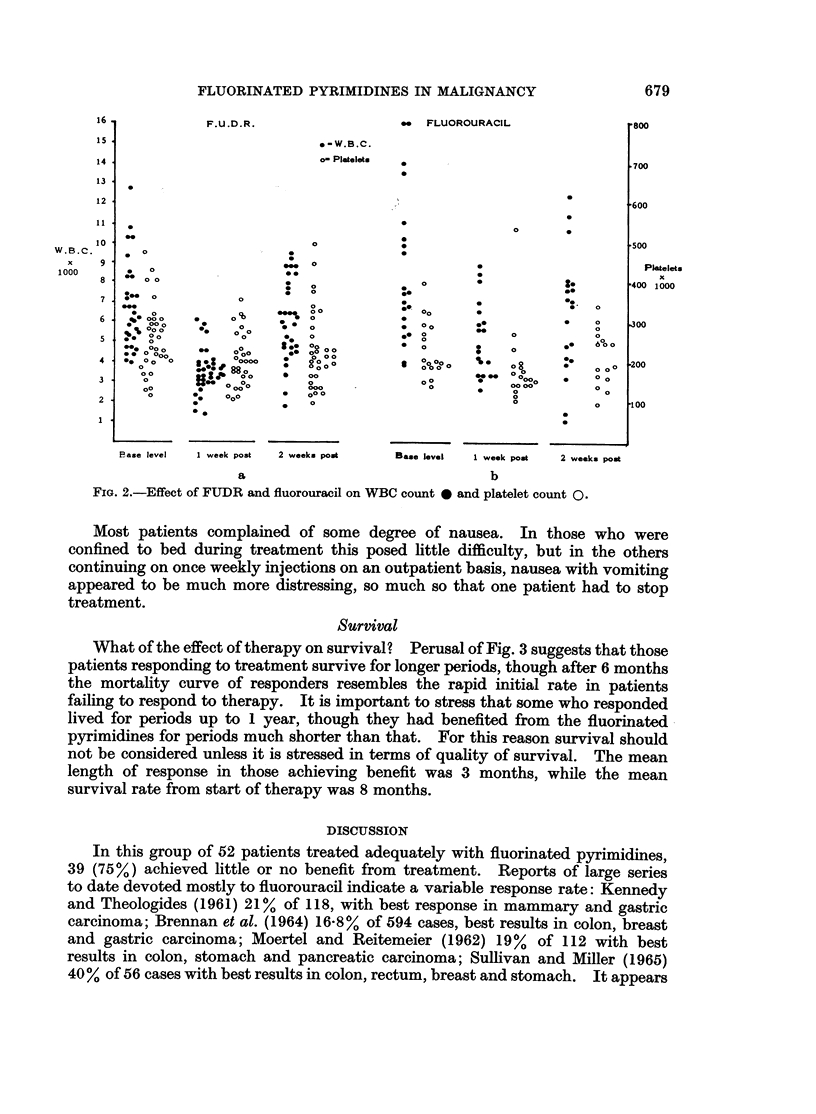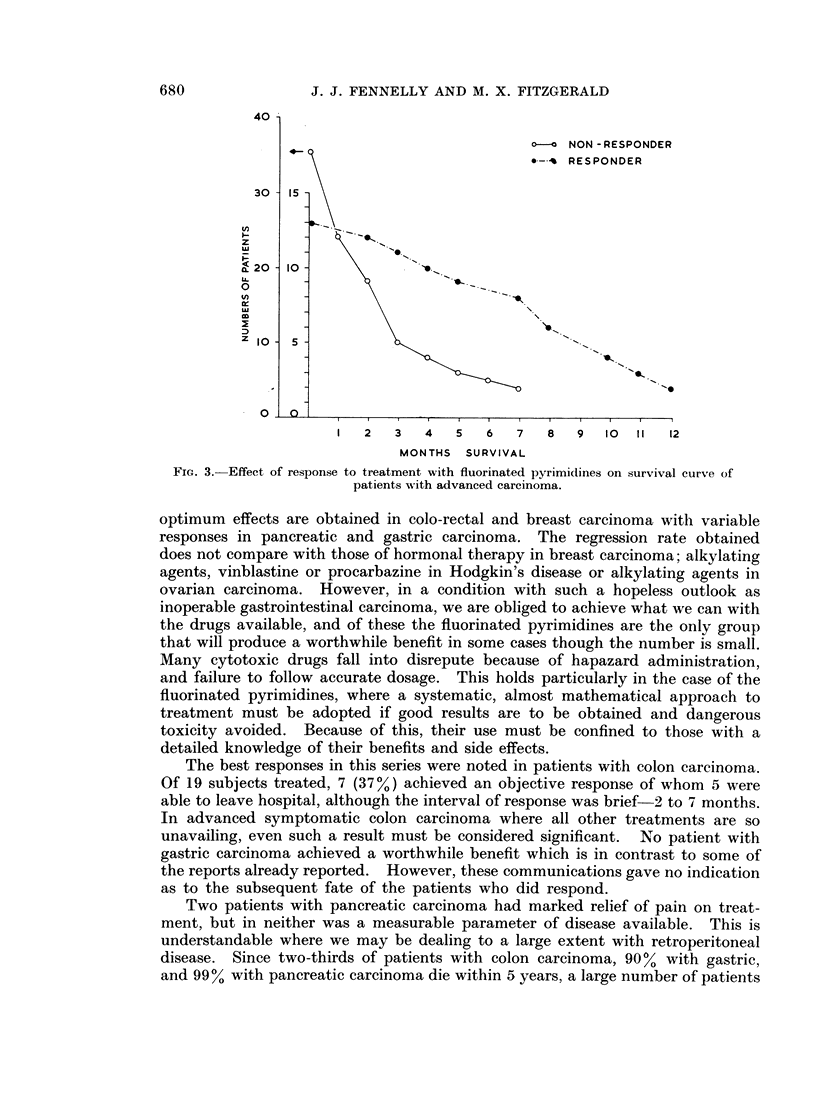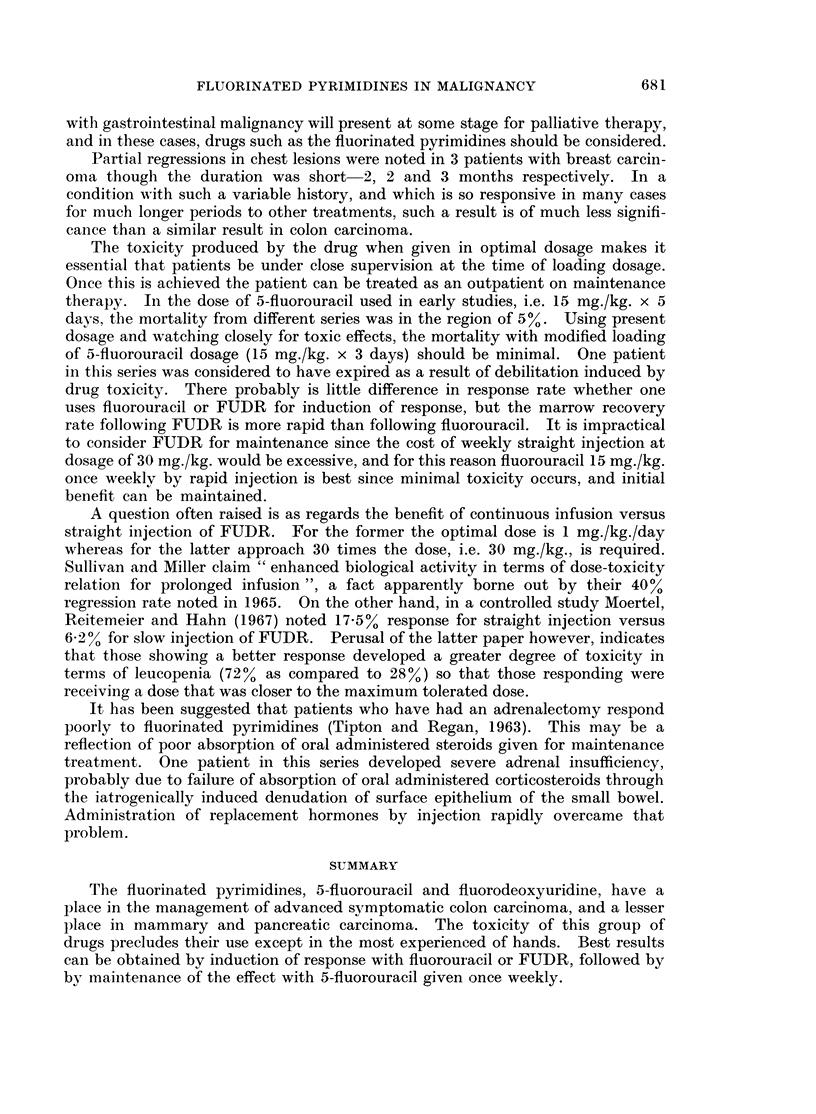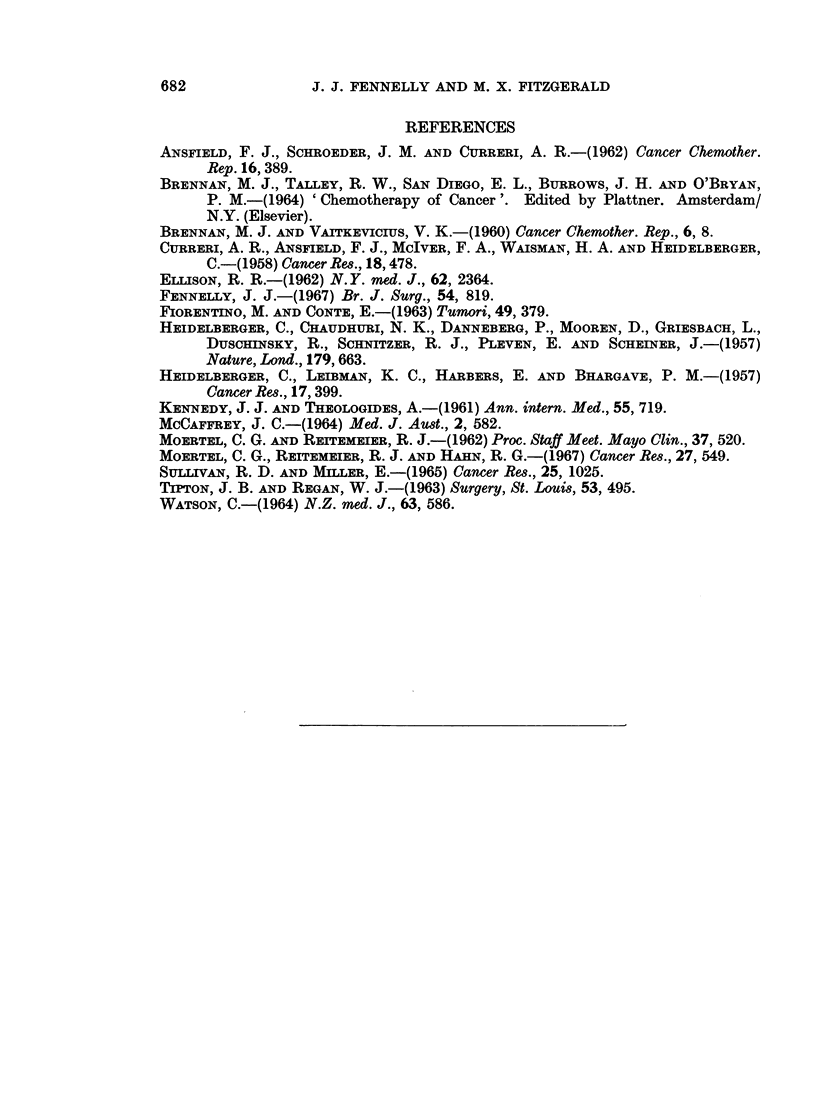# The value of fluorinated pyrimidines in advanced malignancy.

**DOI:** 10.1038/bjc.1968.80

**Published:** 1968-12

**Authors:** J. J. Fennelly, M. X. Fitzgerald


### THE VALUE OF FLUORINATED PYRIMIDINES IN ADVANCED MALIGNANCY

J. J. FENNELLY AND M. X. FITZGERALD

*From the St. Paul's Chemotherapy Unit, Harold's Cross, Box 222, Dublin 6, and the Department of Medicine and Therapeutics, University College, Woodview, Stillorgan Road, Dublin 4*

Received for publication May 10, 1968

THE fluorinated pyrimidines in the form of 5-fluorouracil were introduced to clinical chemotherapy in 1958 by Curreri, Ansfield, McIver, Waisman and Heidelberger. While initial reports might have been highly enthusiastic, larger studies showed an objective regression rate in the region of 20%, though it was felt that this regression rate was achieved only at the expense of high toxicity, and a drug mortality of the order of 5% (Curreri *et al.*, 1958; Moertel and Reitemeier, 1962; Sullivan and Miller, 1965). The one encouraging feature of this group of drugs was that responses were obtained in gastrointestinal malignancy as frequently as in other primaries (Brennan and Vaitkevicos, 1960; Kennedy and Theologides, 1961). Up to that little benefit had been achieved in gastrointestinal adenocarcinoma with the alkylating agents, antibiotics and antimetabolites available. Moertel and Reitemeier's observation (1962) that " 5-fluorouracil must be considered a significant milestone in chemotherapy " appears quite valid. Almost all clinical reports on this drug have come from the United States, except for a few from New Zealand (Watson, 1964), Australia (McCaffrey, 1964) and Italy (Fiorentino and Conte, 1963). Most communications were approached from an experimental investigational point of view, rather than from a strictly therapeutic one. It appears relevant to enquire therefore, why, if a drug is used to such an extent and available commercially on one side of the Atlantic, is it not so accepted on the other side?

The rationale for the use of the fluorinated pyrimidines is based on observations that uracil is incorporated in high concentration into the nucleic acids of rat hepatoma induced chemically, as well as into the intestinal mucosa (Heidelberger, Leibman, Harbers and Bhargave, 1957). Substitution of fluorine in the 5 position of uracil produces a compound, 5-fluorouracil which inhibits thymidilate synthethase, thus preventing conversion of deoxyuridylic acid to thymidylic acid, one of the bases of DNA. This site of action resembles closely one of the sites of action of methothrexate. There is also interference with incorporation of uracil into RNA (Heidelberger, Chaudhuri *et al.*, 1957). Most communications have dealt with fluorouracil, though the riboside fluorodeoxyuridine (FUDR) has been advocated more recently by Ansfield, Schroeder and Curreri 1962.

The purpose of this communication is to report on the treatment of 52 patients with fluorinated pyrimidines (fluorouracil, fluorodeoxyuridine) in an effort to compare results obtained with those two drugs, with emphasis on the real benefit that can be obtained for some patients. Over 90% of the patients treated are now dead, so that it is possible to assess the overall effect of these agents on the life history of the patient with advanced carcinoma.

### MATERIALS AND METHODS

Fifty-two patients with advanced malignancy, who had symptoms requiring treatment, and who had had surgical forms of treatment previously were given adequate courses of either 5-fluorouracil or FUDR as follows: colon 19, stomach 8, pancreas 5, breast 9, ovary 6, lung 2, common bile duct 3. Patients who had reached a terminal state were not included for treatment, nor were they included if there was evidence of bone marrow depression. Only 1 patient over 70 years was treated, since it is generally agreed that this age group tolerates this treatment very poorly. 5-Fluorouracil was given by straight intravenous injection using the modified load dosage recommended by Ellison (1962) as follows: 15 mg./kg./weight straight intravenously daily for 3 days then $7\frac{1}{2}$ mg./kg./day intravenously every second day to toxicity, which usually resulted in 3 to 7 days from the start of treatment. Two weeks after clearing of toxicity maintenance therapy at 15 mg./kg. once weekly was adopted. No patient was given more than 1 g. of fluorouracil in any one injection.

Fluorodeoxyuridine (FUDR) was given by continuous intravenous infusion in dosage of 1 mg./kg./weight, each day's dosage running in over 24 hours to achieve maximal effect (Sullivan and Miller, 1965). This treatment was continued to first signs of toxicity which was usually 2 to 8 days from the start of treatment. If the patient appeared to be responding, and there was no pressing contra-indication on a medical or a social basis, a second loading dose was given 4 weeks later as for the first course. From 2 weeks following complete clearance of toxicity maintenance drug effect was achieved by administration of fluorouracil by rapid intravenous route 15 mg./kg./weight once weekly. In 3 patients with locally recurrent colon neoplasms, FUDR was given by intra-aortic infusion using a Fenwal pressure pump.

### *Toxicity*

Haemoglobin, white cell and platelet counts were checked daily during time of loading dose, and before each weekly maintenance injection. The absolute indications of toxicity leading to cessation of the drug were as follows:

(a) Appearance of oral ulcers—these were sought actively as the patient was not always aware of their presence. Many complained of dryness of the mouth for 24 hours before ulceration appeared.

(b) Diarrhoea, which could not be related to purgatives.

(c) A reduction of white cell count below 4000 c. mm. or platelets below 100,000 c. mm. Relative indication—a sharp drop in white cell count towards a leucopenic level.

### *Responses*

Objective responses were accepted where there was a measurable reduction in disease, associated with improvement in patient's well being lasting over 1 month. Improvement in liver function tests was not accepted alone as evidence of objective response. Subjective responses were accepted where clearance or definite improvement in symptoms occurred on treatment. A category of effect which must be more meaningful from a purely clinical point of view is an improvement of such a degree in a patient admitted requiring treatment, that he or she can return home to carry on their normal routines. Such a phenomenon is not inherent in the objective/subjective rating though it may be a result of such response. As will be seen this occurred in all too few cases.

### RESULTS

The number of patients and their response rate is shown in Table I and details of individual responses are illustrated in Table II.

TABLE I.—*Response of Patients to 5-Fluorouracil and FUDR Treatment*

| | Fluorouracil | | | FUDR | | |
|---|---|---|---|---|---|---|
| | No. | Obj. resp. | Subj. resp. | No. | Obj. resp. | Subj. resp. |
| Colon | 10 | 2 | 2 | 9 | 5* | 1 |
| Stomach | 2 | 0 | 0 | 5 | 0 | 2 |
| Pancreas | 3 | 0 | 1 | 2 | 0 | 1 |
| C.B.D. | 2 | 0 | 0 | 2 | 0 | 0 |
| Lung | — | — | — | 2 | 0 | 1 |
| Breast | 3 | 1 | 0 | 7 | 2 | 0 |
| Ovary | 1 | — | — | 6 | 1 | 1 |
| | 21 | 3 | 3 | 34 | 8 | 6 |

One patient each with mammary, ovarian and bile duct carcinoma was treated with both fluorouracil and fluorodeoxyuridine.

* 2 patients received FUDR by intra-arterial infusion.

*Colon carcinoma* (19)

Nineteen patients received a full course of treatment. Ten received a total of 13 courses of fluorouracil, and 9 patients received 13 full courses of fluorodeoxyuridine. Two of 10 showed an objective response to fluorouracil, both getting such marked relief of pain that they were able to return home.

*Patient* 1 (Table II), a 41 year old housewife obtained marked relief of pain, associated with marked shrinkage of abdominal masses (which had been proved histologically to be neoplastic). The response lasted for 3 months but then complete ureteric obstruction developed secondary to infiltration. It was unfortunate for the patient that a ureterostomy carried out then prolonged her life for 9 months more, since, being resistant to fluorouracil, she continued to require treatment for extremely severe pain.

*Patient* 2, a 50 year old housewife with eqigastric pain and fever due to hepatic metastases, and constipation, showed clearing of all complaints on treatment with fluorouracil in subtoxic dosage.

In both patients the responses lasted only for 3 months, though they lived for 9 and 7 months respectively afterwards with persistent increasing symptoms. Two other patients showed minor symptomatic improvement. Case No. 3 has already been described (Fennelly 1967).

Of the 9 patients treated with FUDR (6 treated systemically and 3 intra-arterially), 5 showed objective and subjective benefit, while one other had definite relief of pain. Three of the total group treated systemically (patients 3, 4 and 5) improved from a stage of incapacitation with pain and other symptoms so they

TABLE II.—*Details of Type of Response Achieved in Those Benefiting from Treatment with Fluorinated Pyrimidines*

| Patient | Dx | Age/ sex | Presenting problem | Drug | Result | Duration of response (months) | Survival from chemotherapy (months) |
|---|---|---|---|---|---|---|---|
| 1. M. M. | Colon | 41 F. | Painful abdom. mass | Fluorouracil | Pain clear; masses 70% reduced | 3 | 12 |
| 2. McL. | ,, | 50 F. | Epigastric pain and fever | ,, | Pain and fever clear | 4 | 9 |
| 3. B.I. | ,, | 55 M. | Pain and fever | FUDR | Pain and fever clear | 6 | 11 |
| 4. J.W. | ,, | 63 M. | Pain and constipation | ,, | Pain cleared | 5 | 12 |
| 5. M.C. | ,, | 64 M. | Pain/bowel obstruction | ,, | Clearing of bowel obstruction and pain | 7 | 8+ |
| 6. C.C. | ,, | 58 M. | Pain/constipation | ,,(I.A.) | Clearing of symptoms mass reduced 40% | 2 | 4 |
| 7. J.R. | ,, | 44 M. | Painful abdom. masses | ,,(I.A.) | Pain + mass reduced 30% | 2 | 2 |
| 8. O.D. | Pancreas | 55 F. | Epigastric pain | ,, | Pain abated | 6 | 7+ |
| 9. N.K. | ,, | 42 F. | Epigastric pain | Fluorouracil | Pain clear | 4 | 8+ |
| 10. P.E. | Breast | 50 F. | Ulcerated chest lesion | ,, | Partial healing of chest wall lesion | 2 | 3 |
| 11. E.F. | ,, | 60 F. | Pain chest recurrence | FUDR | Painful masses reduced in size 20% | 2 | 5 |
| 12. M.B. | ,, | 40 F. | Recurrent chest wall lesions | FUDR | Masses reduced 40% in size | 3 | 10+ |
| 13. M.H. | Lung | 54 F. | Painful hepatic metastases | FUDR | Relief of pain only | 4 | 10 |

I. A. = intra-arterially.

were able to return home for periods of 7, 5 and 7 months respectively. The last of these (Fig. 1) had subacute bowel obstruction at the time of admission, and this cleared completely following 2 courses of FUDR treatment which resulted

FIG. 1.—Response of patient with colon carcinoma to FUDR followed by 5-fluorouracil. WBC count ● and platelet count ○.

also in a 40% reduction in the size of a suprapubic recurrent mass. The response in the patients treated intra-arterially (No. 6 and 7) consisted of shrinkage of recurrent abdominal masses; however, once infusion was stopped there was a rapid recrudescence of lesions and treatment was considered to have been of little real benefit to the patients concerned.

*Pancreas*

Of the 3 patients treated with fluorouracil, one (patient 9) had marked relief of very severe epigastric which lasted for 4 months, but relief was obtained only at the expense of dosage kept to a toxic level, i.e. it was only when diarrhoea was being produced by the drug that real relief of pain was obtained; however, the patient was glad to tolerate this while she experienced relief of pain. One of 2 patients treated with FUDR (patient 8) also obtained good relief of pain though no objective criterion of response was obtainable. Both returned home to live a normal life for 4 to 6 months respectively. One of the remaining 3 obtained minor relief of pain while the remaining 2 cases obtained no benefit.

*Stomach*

Of 7 patients treated, none showed a satisfactory response. It is inherent in this condition that many of the patients at the time of presentation, because of anorexia have lost much weight, and therefore are very unsuitable candidates for any cytotoxic treatment. One patient treated with fluorouracil showed a transient drop in serum bilirubin from 24 to 9·2 mg.% which was not accompanied by any subjective clinical improvement. Two of the 5 patients treated with FUDR noted mild transient relief of pain, but did not improve well enough to leave hospital.

*Lung*

One of the 2 patients with hepatic metastases from lung carcinoma (patient 13) noted relief of pain sufficient to enable her to return home for some months, but there was no change in the size of the liver secondaries, or in persistently abnormal liver function studies.

*Common bile duct*

No improvement resulted in treatment of 3 patients who presented with complete biliary obstruction. Therapy did not affect or delay the rise in serum bilirubin or alkaline phosphatase. No patient presenting with obstructive jaundice as part of other malignant conditions obtained any real benefit.

*Breast*

Of 9 patients treated, 1 responded to fluorouracil, while 2 responded to FUDR. Patient 10, P.E., who had failed to improve following adrenalectomy, showed definite improvement in ulcerative chest wall recurrences, but, at the height of toxicity she developed hyponatraemia with hypotension, probably due to faulty absorption of orally administered replacement corticosteroids, since the serum sodium and blood pressure returned to pretreatment levels following parenteral administration of hydrocortisone. Patients 11 and 12 showed reduction in painful chest wall recurrences for 2 and 3 months respectively, but in terms of the overall survival of these patients this was a small contribution. It is important to point out that all patients in this group had had full hormonal treatment followed by alkylating agents, so they were in a quite advanced state of their disease, when treated with fluorinated pyrimidines.

*Ovary*

One patient with ovarian carcinoma who had developed low grade bowel obstruction showed marked improvement in bowel function while another patient developed partial clearing of a complete bowel obstruction. In neither case was there any measurable objective evidence of improvement. A further patient showed marked reduction in the size of palpable secondaries, but toxicity was of such a degree that no benefit accrued to the patient who died 4 weeks following treatment. While the blood count and bowel function had returned to normal, it is felt that therapy did contribute to the patient's demise.

TABLE III.—*Toxicity Produced by Treatment With 5-Fluorouracil and FUDR*

| | Fluorouracil | FUDR | Total |
|---|---|---|---|
| Oral ulcers | 8/20 (40%) | 9/34 (26%) | 17 (31%) |
| Diarrhoea | 9/20 (45%) | 18/34 (53%) | 27/57 (50%) |
| Leucopenia | | | |
| < 4000 | 9/20 (45%) | 25/34 (74%) | 34/57 (63%) |
| < 3000 | 2/20 (10%) | 11/34 (33%) | 13/57 (22%) |
| < 2000 | 2/20 (10%) | 2/34 (6%) | 4/57 (7%) |
| Thrombocytopenia | | | |
| 100,000 | 1 (5%) | 3/34 (9%) | 4/57 (7%) |

*Toxicity* (*Table* III)

Since the programme adopted here was designed to give full dosage initially, most patients showed some signs of toxicity during the time of loading dosage. This was uncommon in patients on maintenance dosage, occurring only in 4 cases (8%). Fig. 2a and 2b give an outline of the effect of FUDR and 5-fluorouracil respectively on the white cell and platelet counts at the stage of maximum marrow depression and 1 week afterwards. In no case described here was thrombocytopenia a prime reason for ending of treatment. Sixty-six per cent of patients showed a reduction of white cell count below 4000 c. mm. while 4 showed a drop below 2000 c. mm., but in no case did the count go below 1000 c. mm. In almost all cases the white cell count had returned to pretreatment levels within 1 week of cessation of drug therapy, but this return was slower in the case of fluorouracil. Leucopenia was more frequent in those patients on FUDR (70% compared to 40%). Thrombocytopenia produced no clinical problem.

Oral ulceration developed in 31% of cases being more frequent in those treated with fluorouracil. This was of little real discomfort to most subjects, though one patient did develop marked pharyngitis associated with this ulceration. Diarrhoea developed in 50% of cases, a similar frequency in both groups studied. This was usually controlled with Lomotil tablets (diphenoxylate and atropine) combined with kaopectate. In a few cases it was necessary to supplement fluid intake by intravenous infusion.

Alopecia was a clinical problem only in 2 patients, who had developed prolonged granulocytopenia after fluorouracil treatment, though other patients noted increasing " falling out " of hair without cosmetic defects.

FIG. 2.—Effect of FUDR and fluorouracil on WBC count ● and platelet count ○.

Most patients complained of some degree of nausea. In those who were confined to bed during treatment this posed little difficulty, but in the others continuing on once weekly injections on an outpatient basis, nausea with vomiting appeared to be much more distressing, so much so that one patient had to stop treatment.

### *Survival*

What of the effect of therapy on survival? Perusal of Fig. 3 suggests that those patients responding to treatment survive for longer periods, though after 6 months the mortality curve of responders resembles the rapid initial rate in patients failing to respond to therapy. It is important to stress that some who responded lived for periods up to 1 year, though they had benefited from the fluorinated pyrimidines for periods much shorter than that. For this reason survival should not be considered unless it is stressed in terms of quality of survival. The mean length of response in those achieving benefit was 3 months, while the mean survival rate from start of therapy was 8 months.

### DISCUSSION

In this group of 52 patients treated adequately with fluorinated pyrimidines, 39 (75%) achieved little or no benefit from treatment. Reports of large series to date devoted mostly to fluorouracil indicate a variable response rate: Kennedy and Theologides (1961) 21% of 118, with best response in mammary and gastric carcinoma; Brennan *et al.* (1964) 16·8% of 594 cases, best results in colon, breast and gastric carcinoma; Moertel and Reitemeier (1962) 19% of 112 with best results in colon, stomach and pancreatic carcinoma; Sullivan and Miller (1965) 40% of 56 cases with best results in colon, rectum, breast and stomach. It appears

FIG. 3.—Effect of response to treatment with fluorinated pyrimidines on survival curve of patients with advanced carcinoma.

optimum effects are obtained in colo-rectal and breast carcinoma with variable responses in pancreatic and gastric carcinoma. The regression rate obtained does not compare with those of hormonal therapy in breast carcinoma; alkylating agents, vinblastine or procarbazine in Hodgkin's disease or alkylating agents in ovarian carcinoma. However, in a condition with such a hopeless outlook as inoperable gastrointestinal carcinoma, we are obliged to achieve what we can with the drugs available, and of these the fluorinated pyrimidines are the only group that will produce a worthwhile benefit in some cases though the number is small. Many cytotoxic drugs fall into disrepute because of hapazard administration, and failure to follow accurate dosage. This holds particularly in the case of the fluorinated pyrimidines, where a systematic, almost mathematical approach to treatment must be adopted if good results are to be obtained and dangerous toxicity avoided. Because of this, their use must be confined to those with a detailed knowledge of their benefits and side effects.

The best responses in this series were noted in patients with colon carcinoma. Of 19 subjects treated, 7 (37%) achieved an objective response of whom 5 were able to leave hospital, although the interval of response was brief—2 to 7 months. In advanced symptomatic colon carcinoma where all other treatments are so unavailing, even such a result must be considered significant. No patient with gastric carcinoma achieved a worthwhile benefit which is in contrast to some of the reports already reported. However, these communications gave no indication as to the subsequent fate of the patients who did respond.

Two patients with pancreatic carcinoma had marked relief of pain on treatment, but in neither was a measurable parameter of disease available. This is understandable where we may be dealing to a large extent with retroperitoneal disease. Since two-thirds of patients with colon carcinoma, 90% with gastric, and 99% with pancreatic carcinoma die within 5 years, a large number of patients with gastrointestinal malignancy will present at some stage for palliative therapy, and in these cases, drugs such as the fluorinated pyrimidines should be considered.

Partial regressions in chest lesions were noted in 3 patients with breast carcinoma though the duration was short—2, 2 and 3 months respectively. In a condition with such a variable history, and which is so responsive in many cases for much longer periods to other treatments, such a result is of much less significance than a similar result in colon carcinoma.

The toxicity produced by the drug when given in optimal dosage makes it essential that patients be under close supervision at the time of loading dosage. Once this is achieved the patient can be treated as an outpatient on maintenance therapy. In the dose of 5-fluorouracil used in early studies, i.e. 15 mg./kg. × 5 days, the mortality from different series was in the region of 5%. Using present dosage and watching closely for toxic effects, the mortality with modified loading of 5-fluorouracil dosage (15 mg./kg. × 3 days) should be minimal. One patient in this series was considered to have expired as a result of debilitation induced by drug toxicity. There probably is little difference in response rate whether one uses fluorouracil or FUDR for induction of response, but the marrow recovery rate following FUDR is more rapid than following fluorouracil. It is impractical to consider FUDR for maintenance since the cost of weekly straight injection at dosage of 30 mg./kg. would be excessive, and for this reason fluorouracil 15 mg./kg. once weekly by rapid injection is best since minimal toxicity occurs, and initial benefit can be maintained.

A question often raised is as regards the benefit of continuous infusion versus straight injection of FUDR. For the former the optimal dose is 1 mg./kg./day whereas for the latter approach 30 times the dose, i.e. 30 mg./kg., is required. Sullivan and Miller claim " enhanced biological activity in terms of dose-toxicity relation for prolonged infusion ", a fact apparently borne out by their 40% regression rate noted in 1965. On the other hand, in a controlled study Moertel, Reitemeier and Hahn (1967) noted 17·5% response for straight injection versus 6·2% for slow injection of FUDR. Perusal of the latter paper however, indicates that those showing a better response developed a greater degree of toxicity in terms of leucopenia (72% as compared to 28%) so that those responding were receiving a dose that was closer to the maximum tolerated dose.

It has been suggested that patients who have had an adrenalectomy respond poorly to fluorinated pyrimidines (Tipton and Regan, 1963). This may be a reflection of poor absorption of oral administered steroids given for maintenance treatment. One patient in this series developed severe adrenal insufficiency, probably due to failure of absorption of oral administered corticosteroids through the iatrogenically induced denudation of surface epithelium of the small bowel. Administration of replacement hormones by injection rapidly overcame that problem.

### SUMMARY

The fluorinated pyrimidines, 5-fluorouracil and fluorodeoxyuridine, have a place in the management of advanced symptomatic colon carcinoma, and a lesser place in mammary and pancreatic carcinoma. The toxicity of this group of drugs precludes their use except in the most experienced of hands. Best results can be obtained by induction of response with fluorouracil or FUDR, followed by by maintenance of the effect with 5-fluorouracil given once weekly.

### REFERENCES

ANSFIELD, F. J., SCHROEDER, J. M. AND CURRERI, A. R.—(1962) *Cancer Chemother. Rep.* **16**, 389.
BRENNAN, M. J., TALLEY, R. W., SAN DIEGO, E. L., BURROWS, J. H. AND O'BRYAN, P. M.—(1964) 'Chemotherapy of Cancer'. Edited by Plattner. Amsterdam/N.Y. (Elsevier).
BRENNAN, M. J. AND VAITKEVICIUS, V. K.—(1960) *Cancer Chemother. Rep.*, **6**, 8.
CURRERI, A. R., ANSFIELD, F. J., McIVER, F. A., WAISMAN, H. A. AND HEIDELBERGER, C.—(1958) *Cancer Res.*, **18**, 478.
ELLISON, R. R.—(1962) *N.Y. med. J.*, **62**, 2364.
FENNELLY, J. J.—(1967) *Br. J. Surg.*, **54**, 819.
FIORENTINO, M. AND CONTE, E.—(1963) *Tumori*, **49**, 379.
HEIDELBERGER, C., CHAUDHURI, N. K., DANNEBERG, P., MOOREN, D., GRIESBACH, L., DUSCHINSKY, R., SCHNITZER, R. J., PLEVEN, E. AND SCHEINER, J.—(1957) *Nature, Lond.*, **179**, 663.
HEIDELBERGER, C., LEIBMAN, K. C., HARBERS, E. AND BHARGAVE, P. M.—(1957) *Cancer Res.*, **17**, 399.
KENNEDY, J. J. AND THEOLOGIDES, A.—(1961) *Ann. intern. Med.*, **55**, 719.
McCAFFREY, J. C.—(1964) *Med. J. Aust.*, **2**, 582.
MOERTEL, C. G. AND REITEMEIER, R. J.—(1962) *Proc. Staff Meet. Mayo Clin.*, **37**, 520.
MOERTEL, C. G., REITEMEIER, R. J. AND HAHN, R. G.—(1967) *Cancer Res.*, **27**, 549.
SULLIVAN, R. D. AND MILLER, E.—(1965) *Cancer Res.*, **25**, 1025.
TIPTON, J. B. AND REGAN, W. J.—(1963) *Surgery, St. Louis*, **53**, 495.
WATSON, C.—(1964) *N.Z. med. J.*, **63**, 586.